# Emergency Department Response in Dealing with Crimean-Congo Hemorrhagic Fever Patients

**Published:** 2018-09

**Authors:** Siavash HAMZEH POUR, Maryam NAJAFI, Nima DANAEI KOIK

**Affiliations:** 1. Dept. of Pathobiology, School of Public Health, Tehran University of Medical Sciences, Tehran, Iran; 2. Trauma Research Center, Kashan University of Medical Sciences, Kashan, Iran; 3. Dept. of Medical Entomology and Vector Control, School of Public Health, Tehran University of Medical Sciences, Tehran, Iran

## Dear Editor-in-Chief

Crimean-Congo hemorrhagic fever (CCHF) is a febrile hemorrhagic acute disease between animals and humans (Zoonoses), first reported in the Crimea, Ukraine, in 1944 and then in the Congo in 1954. Since then, the name of Crimean-Congo has been used for the virus (1, 2). CCHF is endemic and outbreaks have been recorded in many countries in Africa, the Middle East, Eastern Europe and Asia (3). The Crimean-Congo hemorrhagic fever virus (CCHFV) causes disease and belongs to the genus *Nairovirus* from the family Bunyaviridae. The genome of this virus is a single-stranded RNA virus with a negative polarity (4).

The CCHF disease can be transmitted via the bite of an infective adult tick, particularly *Hyalomma marginatum* or *H. anatolicum* and it can also be transmitted from infected animals and humans (4).

The disease was accompanied by a mild fever in animals, improved by itself after a short fever, but the incubation period in humans is dependent on the mode of transmission. As the incubation period in transmission via bites of infected ticks lasts between 1–3 days and this period in transmission through the blood and tissues takes 6–12 days.

Clinical manifestations of the disease include two stages: non-hemorrhagic and hemorrhagic stages. In the non-hemorrhagic stage, early symptoms of the disease including sudden onset of fever, weakness, severe pain in extremities, back pain, headache, anorexia, and photophobia were observed. In the hemorrhagic and bleeding stage, the symptoms, including bleeding from the gums, nose, leg and hand skin, lung, stomach, and intestine, usually appear four days after the onset of the disease. Severe leukopenia and thrombocytopenia can be seen particularly at this stage. The majority of the patients die from the shock, anemia, excessive bleeding and disseminated intravascular coagulation (DIC). The mortality rate of the disease is usually between 2% and 50% and the disease epidemic in hospital is more common, usually spread by contact with infected blood and body fluids or contaminated needles pushed through the skin (5–7).

Rapid detection of the disease is necessary for monitoring and early treatment of the patients with specific antivirals such as ribavirin and supportive measures, preventing hospital epidemics and bioterrorism attacks of the CCHF virus. To identify the suspected cases of CCHF, biosafety level 4 (BSL4) laboratories are needed. Because in these laboratories specific IgM and IgG antibodies of the patients can be detected using the serological tests such as the ELISA method and also the isolation and sequencing of the genome can be tested by the reverse transcriptase-polymerase chain reaction (RT-PCR) (7,8).

Emergency personnel according to the emergency first aid priorities and before the official confirmation by the Arbovirus Laboratory of the Pasteur Institute and the Center for Infectious Diseases, Ministry of Health and Medical Education may attempt to treat patients without observing protective measures when dealing with the patients with the CCHF virus. Although the knowledge and awareness of the majority of physicians and nurses from the nature of the disease, modes of transmission and symptoms of the disease are relatively high, their knowledge is weak about the system of patient care, treatment of confirmed and suspected cases, as well as reporting of the cases of the disease.

Some cases of this virus and hospital outbreaks were reported in our country and there is no certain vaccine for this disease, necessary protective measures, including wearing gloves, gowns, head coverings, shoe covers, face covers, mask N95, surgical mask and protective glasses should be observed when dealing with persons suspected of having the CCHF disease. Also, transport of infectious specimens from suspected cases of the virus should be performed according to the particular protocol associated with the transport of high-risk infectious specimens (9, 10).
